# Perinatal Depression, Birth Experience, Marital Satisfaction and Childcare Sharing: A Study in Russian Mothers

**DOI:** 10.3390/ijerph18116086

**Published:** 2021-06-04

**Authors:** Vera Yakupova, Liudmila Liutsko

**Affiliations:** 1 Psychological Institute, Russian Academy of Education, 125009 Moscow, Russia; 2 Faculty of Psychology, Lomonosov Moscow State University, 125009 Moscow, Russia; liudmila_liutsko@yahoo.es

**Keywords:** prenatal depression, postnatal depression, perinatal disorders, birth satisfaction, marital satisfaction

## Abstract

Background: Over 300,000 women in Russia face perinatal depressive disorders every year, according to the data for middle-income countries. This study is the first attempt to perform a two-phase study of perinatal depressive disorders in Russia. The paper examines risk factors for perinatal depressive symptoms, such as marital satisfaction, birth experience, and childcare sharing. Methods: At 15–40 gestational weeks (M = 30.7, SD = 6.6), 343 Russian-speaking women, with a mean age of 32 years (SD = 4.4), completed the Edinburgh Postnatal Depression Scale, Couples Satisfaction Index, Birth Satisfaction Scale, and provided socio-demographic data. Two months after childbirth, 190 of them participated in the follow-up. Results: The follow-up indicated that 36.4% of participants suffered from prenatal depression and 34.3% of participants had postnatal depression. Significant predictors of prenatal depression were physical well-being during pregnancy (*β* = −0.25; *p* = 0.002) and marital satisfaction during pregnancy (*β* = −0.01; *p* = 0.018). Birth satisfaction (*β* = −0.08; *p* = 0.001), physical well-being at two months after delivery (*β* = −0.36; *p* < 0.01), and marital satisfaction during pregnancy (*β* = 0.01; *p* = 0.016) and after delivery (*β* = −0.02; *p* < 0.01) significantly predicted postnatal depression at 2 months after delivery. Conclusion: Our study identified that physical well-being during pregnancy and marital satisfaction during pregnancy significantly predicted prenatal depression. Birth satisfaction, physical well-being at 2 months after delivery, and marital satisfaction during pregnancy and after delivery significantly predicted postnatal depression. To our knowledge, this is the first study of perinatal depressive disorders in the context of marital satisfaction and birth satisfaction in the Russian sample. The problem of unequal childcare sharing is widely spread in Russia. Adjusting spousal expectations and making arrangements for childcare may become the focus of psychological work with the family. The availability of psychological support during pregnancy and labor may be important in the context of reducing perinatal depression risks.

## 1. Introduction

Perinatal depression is highly prevalent in low-income and middle-income countries, affecting approximately 25% of women during pregnancy, and about 19% of women after childbirth [1]. It translates to over 300,000 women in Russia facing perinatal depressive disorders each year. Women suffering from prenatal and postnatal depression are at risk for suicide [2], eating disorders, and body dissatisfaction [3]. They tend to have lower self-efficacy and poor self-esteem [4]. Prenatal depression is a risk factor for premature births, low birth weights, and negatively correlates with the length of breastfeeding [5,6]. Furthermore, prenatal depression is a significant predictor of postpartum depression [7]. Depression during pregnancy and after delivery has a negative impact on the development of the emotional and social intelligence of the offspring [8,9,10,11]. Moreover, it may endanger not only the mother’s life but also the health and life of her child [12]. There are risk factors predicting higher rates of prenatal and postnatal depression, such as a history of depressive episodes [7], experience of childhood abuse [13], hypothalamic-pituitary-adrenal dysregulation [14], and physical health problems during pregnancy [15]. In this study, we focused on marital satisfaction and birth experience, because Russia has some specific features concerning family life and delivery practices. According to the Russian Federal Agency for Statistics, most couples who divorce and have children do so in the first years of their child’s life [16]. Today, Russia is transitioning from the extended family to the nuclear one. Most young families with small children used to live in a parental home 20 years ago, whereas today the number of young families living independently is on the rise [17]. Childcare responsibilities are shared between the spouses rather than members of the extended family. According to data from the Ministry of Labor of the Russian Federation, only 2% of fathers take paternity leave to take care of their children. Thus, it is primarily the mother who takes maternity leave and bears the main burden of childcare, which may affect both the quality of the marital relationship and the emotional well-being of the mother [18]. 

However, there is a growing generation of “involved fathers” who participate in childcare from the birth of the child [19]. Studies show marital satisfaction to be a strong factor in reducing the risk of depression during pregnancy and after childbirth [20,21,22]. This is true not only for women but also for men [23]. 

There is evidence linking experiences of loneliness and partner dissatisfaction to depression symptoms during pregnancy [24]. An ambivalent attachment type and constant relationship anxiety are highly correlated with the risk of developing depression after childbirth [16]. 

Birth experience has been shown to be an important factor associated with the risk of postnatal depression [25]. A traumatic birth experience with intensive pain is associated with higher postpartum depression risk [26]. In Russia, most births take place in a maternity hospital solely in the presence of hospital staff, and unaccompanied by a partner or individual assisting specialists [27]. 

The presence of a partner or other family member during labor was not allowed in state-run hospitals until 2012. In many Russian cities, state maternity hospital management is still against doula or personal midwife labor assistance. A conservative Soviet approach is still widely spread and includes a paternalistic style of communication, a lack of ethical concern, outdated medical practices, and the extended medicalization of birth [28]. 

Research into perinatal mental disorders and their interrelations with marital satisfaction, the childcare sharing between partners, and birth satisfaction in Russia is scarce. To our knowledge, this is the first study to address prenatal depressive disorders in a Russian sample. 

The aim of this research is to explore the risk factors for prenatal and postnatal depression, such as satisfaction with the birth process, marital satisfaction during pregnancy and after delivery, planned childcare sharing during pregnancy, and real childcare sharing after delivery, and the adjustment to physical well-being both during pregnancy and after delivery. 

## 2. Materials and Methods

### 2.1. Phases of the Study

The first phase of the study (T1) included screening during pregnancy. The inclusion criteria were as follows: aged over 18 years, a pregnancy at 15 to 40 weeks of gestation, and the ability to read and speak the Russian language. Such a dispersion in the gestational age was chosen to explore the possible associations between the gestational age and risk for prenatal depression, postnatal depression, and marital satisfaction during pregnancy and after delivery. The gestational period of 15 weeks was chosen as the period when a woman can start feeling the baby’s movements and contacting with it [29]. 

The second phase of the study (T2) included the follow-up screening of the participants 2 months after delivery. This time-point was chosen because postnatal symptoms tend to develop during the first months after delivery [30]. The same set of questionnaires was sent to all of the participants who took part in the first phase of the study. Those who experienced antenatal or neonatal loss were excluded from the study (*n* = 1). 

### 2.2. Recruitment of the Participants

The data collection lasted from June 2018 to February 2019. Information about the study was placed in thematic online and offline communities and courses for parents-to-be and new parents. Women who were interested in the participation left their contact information and received an invitation to take part in the study by e-mail. They confirmed the terms of participation in the online form and filled out the questionnaires in the online form as well. Participants demonstrating high scores for perinatal depression were informed about the options to acquire psychological support.

### 2.3. Sample

In the first phase (T1) of the study, 343 women took part and 190 of them participated in the follow-up 2 months after delivery (T2). Of the participants, 100% were Caucasian, spoke the Russian language, and lived in big cities (population over 500,000). The detailed characteristics of the sample are shown in Table 1.

### 2.4. Data Collection Tools

The Russian version of the Edinburgh Postnatal Depression Scale (EPDS) [31] was used to measure the intensity of pre- and postnatal depression. It is a 10-question scale that indicates how the mother has felt during the previous week. A 4-point Likert scale is used for each question. A score of 10 and higher is suggested to indicate possible depression (Cronbach’s α = 0.838). 

The Couples Satisfaction Index (CSI) is a 16-item questionnaire that is used to measure satisfaction in a relationship with four subscales (partner’s support, respect, the pleasure of interaction, quality of relationships) [32]. A 6-point Likert scale is used for each question, the last block is a semantic differential scale with 6-point rating options. A total score lower than 50 may indicate relationship dissatisfaction (Cronbach’s α = 0.817). 

The participants were asked to measure their expectations of childcare sharing with their partner in percentages (0–100%, where 100% is all of the baby’s needs) in answer to the following question: “How much of the childcare do you plan to share with the father of the child?” We asked the participants to assess their state of physical well-being at the time of the first screening during pregnancy using a 5-point Likert scale (1 = “very poor”, 5 = “very good”). We also collected data on the socio-demographics, the number of children, etc.

At the second stage of the study, we repeated the questionnaires used during the first phase and, in addition, we used the following ones. A Birth Satisfaction Scale-Revised Indicator (BSS-RI)-short 6-item self-report questionnaire to assess birth satisfaction (the subscales are the level of stress and anxiety, feeling of control, medical staff support) [33]. A 3-point Likert scale is used for each question (range 0–2). While higher scores represent greater birth satisfaction, a total score lower than 50 is supposed to show relationship dissatisfaction (Cronbach’s α = 0.701). We asked the participants to assess their state of health at the time of the second screening (2 months after delivery) using a 5-point Likert scale (1 = “very poor”, 5 = “very good”). The participants were asked to measure the childcare sharing with the partner in percentages in answer to the following question: “How much of the childcare do you share with the father of the child?”. We asked the participants about the mode of delivery and type of delivery support, place and time of delivery, and gestational age.

### 2.5. Data Analysis

The main variables of the study were prenatal and postnatal depression (EPDS), marital satisfaction during pregnancy and after delivery (CSI), childcare share during pregnancy and after delivery, physical well-being during pregnancy and after delivery, and birth satisfaction (BSSR-RI). 

The Mann–Whitney U-test was used to examine the difference between the group that took the invitation to participate in the second phase of the study and the group that did not answer the invitation. Spearman’s correlation coefficient was used to measure the associations between pre- and postnatal depression, marital satisfaction, birth satisfaction, physical well-being during pregnancy and after delivery, and childcare sharing. 

A dependent sample *t*-test was used to describe the changes in the level of depression, childcare sharing, and marital satisfaction during pregnancy and after delivery. In order to examine the factors predicting prenatal and postnatal depression, we conducted linear regression analysis. The dependent variable in model one was prenatal depression, and the independent variables were gestational age, marital satisfaction during pregnancy, physical well-being during pregnancy, and maternal planned childcare share, whilst controlling for age also. The dependent variable in model two was postnatal depression (EPDS scores), and the independent variables were marital satisfaction during pregnancy and after delivery, physical well-being 2 months after delivery, gestational age at birth, birth satisfaction, and maternal real childcare share, whilst controlling for age also.

We used the Kruskal–Wallis test to assess the differences in the levels of prenatal and postnatal depression between the groups with different forms of labor assistance (alone, with a partner, with a doula or a midwife and a partner, and with a doula or a midwife without a partner) and the mode of delivery. All analyses were performed using SPSS Statistics 22 (IBM SPSS Statistics, Russian Federation).

### 2.6. Ethical Consideration

This study was conducted in accordance with the recommendations of the Declaration of Helsinki. The protocol was approved by the Ethical Committee of the Russian Psychological Society. The study was approved by the Ethics Committee of the Faculty of Psychology at Lomonosov Moscow State University (approval code #18/1102). 

## 3. Results

In the first phase of the study (T1), 36.4% (*n* = 125) of participants had prenatal depression. At the follow-up (T2), 34.3% (*n* = 65) of participants had postnatal depression. 

The women who responded to the invitation to take part in the follow-up (T2) showed lower depression levels during pregnancy (U = 12.179, *p* = 0.007) in comparison to the women who took part in the first phase of the study (T1) but did not respond to the invitation to participate in the follow-up two months after delivery (T2). No statistically significant differences in age, gestational age, education level, or marital satisfaction level were found between the two groups. 

The levels of depression did not differ significantly 2 months after delivery compared to that during pregnancy (t = −0.557, *p* = 0.568). No statistically significant correlations were found between the gestational age and the levels of prenatal and postnatal depression and marital satisfaction before and after delivery. The descriptive statistics of the main variables during T1 and T2 of the study are shown in Table 2. 

### 3.1. Satisfaction with the Birth Process and Perinatal Depression

Satisfaction with the birth process negatively correlates with postnatal depression (rho = −0.28, *p* < 0.01). No statistically significant relationships were found to exist between the delivery modes, vaginal birth (VB), emergency C-section (ECS), and elective C-section (ELCS), and the level of pre- and postpartum depression (H = 2.444, *p* = 0.295; H = 0.033, *p* = 0.983). The groups based on birth support (partner, doula or private midwife, no support) did not differ significantly in the levels of prenatal and postnatal depression (H = 1.464, *p* = 0.691; H = 3.270, *p* = 0.352).

### 3.2. Marital Satisfaction and Perinatal Depression

Marital satisfaction significantly decreased 2 months after delivery compared to that during pregnancy (t = 4.429, *p* < 0.001). We found a significant correlation between marital satisfaction and after delivery (rho = −0.43, *p* < 0.01).

During pregnancy, the expected childcare share was 70% for the mother and 30% for the father (when 100% means all of the baby’s needs). After childbirth, the woman’s real burden proves to be higher, averaging at about 80% (t = −6.572, *p* < 0.001), and the man’s burden proves lower, at about 20% (t = −6.296, *p* < 0.001). After delivery, a higher planned mother’s childcare share and real maternal childcare share are associated with higher rates of depression (rho = 0.195, *p* < 0.01; rho = 0.219, *p* < 0.01).

### 3.3. Physical Well-Being and Perinatal Depression

The severity of prenatal depression and postpartum depression symptoms is associated with lower scores of well-being during pregnancy (rho = −0.20, *p* < 0.01; rho = −0.18, *p* < 0.01. Lower scores of postpartum depression correlate with a higher quality of well-being 2 months after delivery (rho =−0.39, *p* < 0.01).

### 3.4. Linear Regression Analysis for Predicting Prenatal and Postnatal Depression

We performed a linear regression including prenatal depression as a dependent variable. Physical well-being during pregnancy, gestational age, marital satisfaction, and planned childcare sharing were the independent variables (see Table 3). The analysis revealed that these variables contributed significantly to the regression model F (5, 143) = 10.96, *p* < 0.001 and accounted for 27.7% of the variance in prenatal depression. The significant predictors of prenatal depression were physical well-being (*β* = −0.25; *p* = 0.002) and marital satisfaction during pregnancy (*β* = −0.01; *p* = 0.018).

The second linear regression model included postnatal depression as a dependent variable, and marital satisfaction during pregnancy and after delivery, real maternal childcare share, prenatal depression, gestational age at birth, birth satisfaction and physical well-being two months after delivery as independent variables. These variables contributed significantly to the regression model F (7, 176) = 13.64, *p* < 0.001 and accounted for 35.2% of the variance in postnatal depression. The significant predictors of postnatal depression were marital satisfaction during pregnancy (*β* = 0.013; *p* = 0.016), marital satisfaction after delivery (*β* = −0.022; *p* < 0.001), physical well-being 2 months after delivery (*β* = −0.362; *p* < 0.001), and birth satisfaction (*β* = −0.075; *p* = 0.001).

## 4. Discussion

Our study identified that physical well-being during pregnancy and marital satisfaction during pregnancy significantly predicted prenatal depression. Birth satisfaction, physical well-being 2 months after delivery, and marital satisfaction during pregnancy and after delivery significantly predicted postnatal depression. The results of our study in the Russian sample are consistent with other research on the problem and show that perinatal disorders are a complex, multi-component phenomenon [21,34]. In our study, 36.4% of the participants had prenatal depression and 34.3% of the participants had postnatal depression, which seems to be higher than the statistics for middle-income countries [1].

Studies show a decrease in marital satisfaction after childbirth [35,36]. The data obtained in our research showed an unexpected result in the linear regression model for postnatal depression. Higher levels of marital satisfaction during pregnancy predicted higher postnatal depression scores. It turns out that the women who had high CSI scores during pregnancy and low CSI scores after delivery are at higher risk of postnatal depression. There is a possibility that, for these women, the negative changes in the partner relationships themselves are a factor for depression development, rather than the low marital satisfaction after delivery. The changes in marital satisfaction and their relations with depression after delivery might be a direction for further research investigation could also be carried out regarding The topic of the further research could concern the Couples’ Satisfaction index. Six points of each item might reflect the idealization of marriage. For example: 1. “Please, indicate the degree of happiness of your relationship”, the six-score answer is “Perfect”. 2. “In general, how often do you think that things between you and your partner are going well?”, the six-score answer is “All the time”. We can assume that women with higher scores tend to idealize their marriage during pregnancy and experience more hard feelings about the changes in their marital relationship after delivery.

In our study, a father’s actual involvement in childcare is far from being equal and is short of the mother’s expectations. The childcare share variable did not show significant associations with prenatal and postnatal depression in the linear regression models. However, it is associated with marital satisfaction during pregnancy and after delivery. The traditional distribution of childcare duties (when a mother is the main caregiver) is still widely spread [37], but our data suggests that it no longer satisfies the expectations of women with higher education living in big cities. The study of parental burnout in 42 countries [38] shows that Russia is approaching Europe and North America in the growing trend of “intensive parenting”. The demands on the parent are high, the support of the extended family is low. In these new circumstances, the balance of childcare needs revision. It may be important to perform more detailed research on the variable of the childcare share, for example, by adding a question about the mother’s satisfaction with the distribution of the childcare duties and about other members of the family taking part in childcare.

Adjusting spousal expectations and making arrangements for childcare may become the focus of psychological work with the family before and during pregnancy [39,40]. Psychological education and work on the quality of relationships in couples are generally associated with greater marital satisfaction after delivery, empathy, and mutual support [41]. 

Our findings are consistent with evidence that psychologically and physically traumatic childbirth experiences may become a serious risk factor for postpartum depression [25,34,42]. There is an increasing number of partner births in Russia. According to our results, the rates of birth alone at the hospital and partner birth are practically equal. Partner birth may help enhance relationship functioning and partner involvement in childcare [43,44]. There is evidence that men who were present at the delivery are more likely to show empathy and emotional support for their spouse [45]. Higher birth satisfaction significantly predicts lower postpartum depression scores 2 months after delivery. Hospital administrations start focusing on psychological comfort and support availability during labor, but this process is still at its very beginning [46]. A major issue during childbirth in Russia today is partner accompaniment and assistance from specialists, such as an individual midwife or a doula, at childbirth because these practices are far from being typical of every city and maternity hospital.

## 5. Conclusions

Our study identified that physical well-being during pregnancy and marital satisfaction during pregnancy predicted prenatal depression significantly. Birth satisfaction, physical well-being 2 months after delivery, and marital satisfaction during pregnancy and after delivery significantly predicted postnatal depression. To our knowledge, this is the first study of perinatal depressive disorders in the context of marital satisfaction and birth satisfaction in the Russian sample. The problem of unequal childcare sharing is widely spread in Russia. Adjusting spousal expectations and making arrangements for childcare may become the focus of psychological work with a family as early as it plans and expects a child. The study results raise the question of the importance of psychological support for the mother during pregnancy, especially if the woman is experiencing issues with her physical well-being. The availability of support and psychological comfort during labor may be crucial in the context of reducing postpartum depression risks.

Study Limitations and future research: One of this study’s limitations is that we used only self-reporting techniques that are less reliable in comparison to clinical examinations. It is important to mention that 45% of participants dropped out of the study and did not take part in the follow-up. This resulted in biased data, since women with lower levels of prenatal depression tended to participate in the second phase of the study. Women with higher rates of prenatal depression might be in a less resourceful state after delivery and less willing to take part in the next phase of the study. The gestational age from 15 to 40 weeks of pregnancy is wide and may also result in data biases. The construct of childcare sharing needs further detailed research. Our data was not normally distributed and does not allow us to drive the conclusions about the prevalence of perinatal depression in the Russian population. In addition, the sample size of this study does not allow us to perform analysis by group (levels of EPDS and/or CSI) to detect significant effects in some subgroups. For these reasons, to explore the effects in detail, further studies are needed with a larger sample size. More studies are needed on the effects of this interventional work prior to pregnancy on the overall well-being of women during pregnancy and after delivery.

## Figures and Tables

**Table 1:** Characteristics of the sample.

		T1 (*n* = 343)			T2 (*n* = 190)			
		M/N	±SD/%	Range	M/N	±SD/%	Range	*p*-Value
Age at testing (years)		32	4.4	19–46	32	4.3	19–46	
Education	Upper secondary/College	32	9.3%		16	8.4%		
	Tertiary/University	311	90.7%		174	91.6%		
Family status	Married	284	82.8%		158	83%		
	Cohabiting with a partner	49	14.3%		27	14.5%		
	Single	10	2.9%		5	2.5%		
Time passed after the childbirth (months)					1.9	0.22	1.7–2.1	
Gestation week		30.7	6.6	15–40				
Week of birth					39.4	1.6	37–42	
Parity	primiparous	139	40.4%	82	43%			
	multiparous	204	59.6%	108	57%			
Delivery mode	Vaginal				139	73%		
	Emergency Cesarean				23	12%		
	Elective Cesarean				28	15%		
Mode of birth support	No support				69	36.1%		
	Partner				75	39.3%		
	Doula/Private midwife				15	8.2%		
	Partner + Doula/Private midwife				8.2%	16.4%		
EPDS score		8.5	5.4	0–28	7.9	2.3	0–21	*p* = 0.568
CSI score		62.4	17.3	0–81	60.4	17.9	0–81	*p* < 0.001
Physical well-being during pregnancy/2 months after delivery		3.6	0.9	1–5	4.2	0.8	2–5	*p* < 0.001
Maternal share of childcare planned/real		71.9	14.4	0–100	78.9	14.4	40–100	*p* < 0.001
BSSR-RI score					7.3	2.2	0–10	

Legend: EPDS, postnatal depression (measured using the Edinburgh Postnatal Depression Scale); CSI, marital satisfaction (measured using the Couples Satisfaction Index); BSS-RI, birth satisfaction (measured using the Birth Satisfaction Scale Revised). Where *p* shows statistical differences between the first phase of the study (T1) (*n* = 343) and the follow-up (T2) (*n* = 190).

**Table 2:** Correlations between the main variables of the study.

	EPDS T2	BSSR-RI	Physical Well-Being T1	Physical Well-Being T2	CSI T1	CSI T2	Maternal Planned Childcare Share	Maternal Real Childcare Share
EPDS T1	0.48 **	0.01	−0.20 **	−0.08	−0.04	−0.07	−0.02	−0.034
EPDS T2		−0.28 **	−0.18 **	−0.39 **	−0.25 **	−0.43 **	0.19 **	0.22 **
BSSR_RI				0.07	0.23 **	0.26 **	−0.16 *	−0.19
Physical well-being T2						0.20 *	−0.08	−0.05
CSI T1						0.80 **	−0.30 **	−0.24 **
CSI T2							−0.32 **	−0.41 **

Legend: rho, ** *p* < 0.01, * *p* < 0.05.

**Table 3:** Linear regression results examining associations with prenatal depression at phase one and postnatal depression at phase two of the longitudinal study.

	β	Std β	R^2^	ΔR^2^
Phase one (Prenatal Depression)				
CSI (pregnancy)	−0.01 *	0.004	0.28	0.25
Physical well-being in the III trimester	−0.246 **	0.8		
Phase two (Postnatal Depression)				
CSI (pregnancy)	0.016 *	0.005	0.35	0.33
CSI (postpartum)	−0.02 ***	0.005		
Physical well-being (2 months after delivery)	−0.36 ***	0.06		
Birth satisfaction	−0.07 **	0.02		

Legend: * *p* < 0.05, ** *p* < 0.01, *** *p* < 0.001.

## Data Availability

The datasets used and/or analyzed during the current study are available from the corresponding author upon request.

## References

[1] Gelaye B., Rondon M.B., Araya R., Williams M.A. (2016). Epidemiology of maternal depression, risk factors, and child outcomes in low-income and middle-income countries. Lancet Psychiatry.

[2] Gressier F., Guillard V., Cazas O., Falissard B., Glangeaud-Freudenthal N.M.-C., Sutter-Dallaye A.-L. (2017). Risk factors for suicide attempt in pregnancy and the post-partum period in women with serious mental illnesses. J. Psychiatr. Res..

[3] Riquin E., Lamas C., Nicolas I., Lebigre C.D., Curt F., Cohen H., Legendre G., Corcos M., Godart N. (2019). A key for perinatal depression early diagnosis: The body dissatisfaction. J. Affect. Disord..

[4] Zheng X., Morrell J., Watts K. (2018). Changes in maternal self-efficacy, postnatal depression symptoms and social support among Chinese primiparous women during the initial postpartum period: A longitudinal study. Midwifery.

[5] Grigoriadis S., VonderPorten E.H., Mamisashvili L., Tomlinson G., Dennis C.L., Koren G., Steiner M., Mousmanis P., Cheung A., Radford K. (2013). The impact of maternal depression during pregnancy on perinatal outcomes: A systematic review and meta-analysis. J. Clin. Psychiatry.

[6] Jarde A., Morais M., Kingston D., Giallo R., MacQueen G.M., Giglia L., Beyene J., Wang Y., McDonald S.D. (2016). Neonatal Outcomes in Women With Untreated Antenatal Depression Compared With Women Without Depression: A Systematic Review and Meta-analysis. JAMA Psychiatry.

[7] McMahon C., Boivin J., Gibson F.L., Hammarberg K., Wynter L., Fisher J. (2015). Older maternal age and major depressive episodes in the first two years after birth: Findings from the Parental Age and Transition to Parenthood Australia (PATPA) study. J. Affect. Disord..

[8] Madigan S., Oatley H., Racine N., Fearon R.M.P., Schumacher L., Akbari E., Cooke J.E., Tarabulsy G.M. (2018). A Meta-Analysis of Maternal Prenatal Depression and Anxiety on Child Socioemotional Development. J. Am. Acad. Child Adolesc. Psychiatry.

[9] Drury S., Scaramella L., Zeanah C. (2016). The Neurobiological Impact of Postpartum Maternal Depression: Prevention and Intervention Approaches. Child Adolesc. Psychiatr. Clin. N. Am..

[10] Burmenskaya G.V. (2009). Child’s Attachment to Mother as the Basis of Mental Development Typology. Psychol. Russ. State Art..

[11] Evans J., Melotti R., Heron J., Ramchandani P., Wiles N., Murray L. (2012). The timing of maternal depressive symptoms and child cognitive development: A longitudinal study. J. Child Psychol. Psychiatry.

[12] Jacques N., Loret de Mola C., Joseph G., Mesenburg M.A., Silveira M.F. (2019). Prenatal and postnatal maternal depression and infant hospitalization and mortality in the first year of life: A systematic review and meta-analysis. J. Affect. Disord..

[13] Wosu A.C., Gelaye B., Williams M.A. (2015). History of Childhood Sexual Abuse and Risk of Prenatal and Postpartum Depression or Depressive Symptoms: An Epidemiologic Review. Arch. Women Ment. Health.

[14] Yim I.S., Stapleton L.R.T., Guardino C.M., Hahn-Holbrook J., Schetter C.D. (2015). Biological and Psychosocial Predictors of Postpartum Depression: Systematic Review and Call for Integration. Annu. Rev. Clin. Psychol..

[15] Pace R., Rahme E., Da Costa D., Dasgupta K. (2018). Association between gestational diabetes mellitus and depression in parents: A retrospective cohort study. Clin. Epidemiol..

[16] Marques R., Monteiro F., Canavarro M.C., Fonseca A. (2018). The role of emotion regulation difficulties in the relationship between attachment representations and depressive and anxiety symptoms in the postpartum period. J. Affect. Disord..

[17] Eliseeva I.I. (2016). Evolution of the Structure of Russian Households: A Tentative Explanation. Petersb. Sociol. Today.

[18] Lin W.-C., Chang S.-Y., Chen Y.-T., Lee H.-C., Chen Y.-H. (2017). Postnatal paternal involvement and maternal emotional disturbances: The effect of maternal employment status. J. Affect. Disord..

[19] Noskova A.V. (2015). New Methodological Approaches, Research Tricks, Discussion Problems of Family Sociology. Sociol. Stud..

[20] Yakupova V.A. (2018). The Impact of Psychological and Physiological Conditions of Motherhood on Postnatal Depression. Russ. J. Psychol..

[21] Yusuff A.S.M., Tang L., Binns C.W., Lee A.H. (2015). Prevalence and risk factors for postnatal depression in Sabah, Malaysia: A cohort study. Women Birth.

[22] Shi P., Ren H., Li H., Dai Q. (2018). Maternal depression and suicide at immediate prenatal and early postpartum periods and psychosocial risk factors. Psychiatry Res..

[23] Da Costa D., Danieli C., Abrahamowicz M., Dasgupta K., Sewitch M., Lowensteyn I., Zelkowitz P. (2019). A prospective study of postnatal depressive symptoms and associated risk factors in first-time fathers. J. Affect. Disord..

[24] Luoma I., Korhonen M., Puura K., Salmelin R.K. (2018). Maternal loneliness: Concurrent and longitudinal associations with depressive symptoms and child adjustment. Psychol. Health Med..

[25] Bell A.F., Andersson E. (2016). The birth experience and women’s postnatal depression a systematic review. Midwifery.

[26] Ponti L., Smorti M. (2019). Mediating Role of Labor on the Relationship Between Prenatal Psychopathologic Symptoms and Symptoms of Postpartum Depression in Women Who Give Birth Vaginally. J. Obstet. Gynecol. Neonatal Nurs..

[27] Borozdina E., Novkunskaya A. (2020). Patient-centered care in Russian maternity hospitals: Introducing a new approach through professionals’ agency. Health Interdiscip. J. Soc. Study Health Illn. Med..

[28] Temkina A.A. (2014). Medicalization of childbirth: Fight for control. J. Soc. Policy Res..

[29] Zakharova E.I. (2016). Developmental psychology and psychological support during the transition to parenting period. World Psychol..

[30] Anokye R., Acheampong E., Budu-Ainooson A., Obeng E.I., Akwasi A.G. (2018). Prevalence of postpartum depression and interventions utilized for its management. Ann. Gen. Psychiatry.

[31] Yakupova V.A., Bukhalenkova D.A. (2019). Prenatal Depression: Risk factors and ways of prevention. Issues Psychol..

[32] Funk J.L., Rogge R.D. (2007). Testing the ruler with item response theory: Increasing precision of measurement for relationship satisfaction with the Couples Satisfaction Index. J. Fam. Psychol..

[33] Martin C.R., Martin H.C., Redshaw M. (2017). The Birth Satisfaction Scale-Revised Indicator (BSS-RI). BMC Pregnancy Childbirth.

[34] Mohammad K.I., Gamble J., Creedy D.K. (2011). Prevalence and factors associated with the development of antenatal and postnatal depression among Jordanian women. Midwifery.

[35] Delicate A., Ayers S., McMullen S. (2018). A systematic review and meta-synthesis of the impact of becoming parents on the couple relationship. Midwifery.

[36] Doss B.D., Rhoades G.K. (2017). The transition to parenthood: Impact on couples’ romantic relationships. Curr. Opin. Psychol..

[37] Temkina A.A. (2010). Childbearing and Work-Family Balance among contemporary Russian women. Finish Yearb. Popul. Res..

[38] Roskam I., Aguiar J., Akgun E., Arikan G., Artavia M., Avalosse H., Aunola K., Bader M., Bahati C., Barham E.J. (2021). Parental Burnout Around the Globe: A 42-Country Study. Affect. Sci..

[39] Shockley K.M., Allen T.D. (2018). It’s not what I expected: The association between dual-earner couples’ met expectations for the division of paid and family labor and well-being. J. Vocat. Behav..

[40] Cho H.J., Kwon J.H., Lee J.J. (2008). Antenatal cognitive-behavioral therapy for prevention of postpartum depression: A pilot study. Yonsei Med. J..

[41] Suto M., Takehara K., Yamane Y., Ota E. (2016). Effects of prenatal childbirth education for partners of pregnant women on paternal postnatal mental health: A systematic review and meta-analysis protocol. J. Affect. Disord..

[42] Dikmen-Yildiz P., Ayers S., Phillips L. (2017). Factors associated with post-traumatic stress symptoms (PTSS) 4-6 weeks and 6 months after birth: A longitudinal population-based study. J. Affect. Disord..

[43] Daley-McCoy C., Rogers M., Slade P. (2015). Enhancing relationship functioning during the transition to parenthood: A cluster-randomised controlled trial. Arch. Women Ment. Health.

[44] Gambrel L.E., Piercy F.P. (2015). Mindfulness-based relationship education for couples expecting their first child–part 1: A randomized mixed-methods program evaluation. J. Marital Fam..

[45] Coutinho E.C., Vilaça J.G., Antunes C., Duartea J.C., Parreirac V.C., Chaves C.M.B., Nelas P.A.B. (2016). Benefits for the Father from their Involvement in the Labour and Birth Sequence. Procedia Soc. Behav. Sci..

[46] Ozhiganova A.A. (2020). Active mistrust of doctors: A case study of legal support during childbirth. Medical anthropology research. Sib. Hist. Res..

